# Traumatic Isolated Avulsion Rupture of the Distal Semitendinosus Tendon in a Non-athlete

**DOI:** 10.7759/cureus.45141

**Published:** 2023-09-12

**Authors:** Abdelrafour Houdane, Rana K Othman, Haroon A Javaid, Abdullah M Taha, Islam M Ahmmed, Ahmed E Maklad

**Affiliations:** 1 College of Medicine, Alfaisal University, Riyadh, SAU; 2 Radiology, Saudi German Hospital, Riyadh, SAU

**Keywords:** rupture, avulsion, tendon, semitendinosus tendon, hamstring tendon, non-athlete, hamstring tendons (semitendinosus and gracilis), distal semitendinosus injury

## Abstract

Isolated distal semitendinosus (ST) injuries remain an uncommon hamstring injury, with avulsion ruptures reported even less frequently. These injuries occur due to eccentric overloading seen in sprinting or jumping injuries. Treatment ranges from conservative management to surgical tenotomy or reattachment to the tibial bone. We present a unique case of a 30-year-old male with an isolated avulsion rupture of the distal ST tendon after a fall. To our knowledge, this is the first case reported in the literature of an isolated distal ST injury in a non-athlete due to trauma.

## Introduction

Hamstring injuries are one of the most commonly encountered muscle injuries sustained by athletes [1]. Rapid acceleration, deceleration, and pivoting forces place a significant demand on the hamstrings and tendons [2], increasing their risk of injury. The mechanism of injury for proximal and distal avulsion injuries is often due to eccentric overload compared to muscle belly strains which are more related to concentric contractions while sprinting [3].

Strains to the muscle or myotendinous junction account for most cases of hamstring injuries [4-6], but distal and proximal hamstring tendon injuries have also been reported in the literature. The biceps femoris remains the most common site of hamstring injury accounting for up to 87% of all hamstring injuries, with semitendinosus (ST) injury accounting for 32% to 37% of injuries [3,7,8]. Isolated ST injuries account for only 10% of all hamstring injuries [8].

A literature search revealed that isolated avulsion rupture of the distal ST is very uncommon, with only a few case reports, and exclusively as an athletic injury during sports [9,10]. This article presents a case of an isolated avulsion rupture of the distal ST tendon of traumatic origin. To our knowledge, this is the first reported case of a distal ST injury in a non-athlete and secondary to trauma.

## Case presentation

A 30-year-old male presented to our institution with severe pain in the upper left calf region. The patient had a history of anterior cruciate ligament (ACL) reconstruction with a non-ST graft performed approximately two years prior in the same limb. He was otherwise in good health. The patient sustained direct trauma to the mentioned region due to a fall down the stairs, which was associated with a popping sound along the posteromedial aspect of the left knee.

On clinical examination, the patient’s findings consisted of edema in the upper left calf region, localized tenderness, and ecchymosis. The patient also displayed hardening of the calf muscles and pain with plantar flexion, leading to a provisional diagnosis of plantaris tendon rupture based on the clinical picture. The ST tendon was not visible or palpable on physical examination, and following the tendon’s path on ultrasound showed a distal avulsion injury of the ST tendon.

The patient demonstrated a normal gait cycle with symmetry and smoothness, with intact knee and ankle reflexes. Assessment of joint function through passive and active movements revealed an unaffected range of motion. There were no concerns regarding knee stability, and Simmonds’ test ruled out rupture of the Achilles tendon.

**Figure 1:**
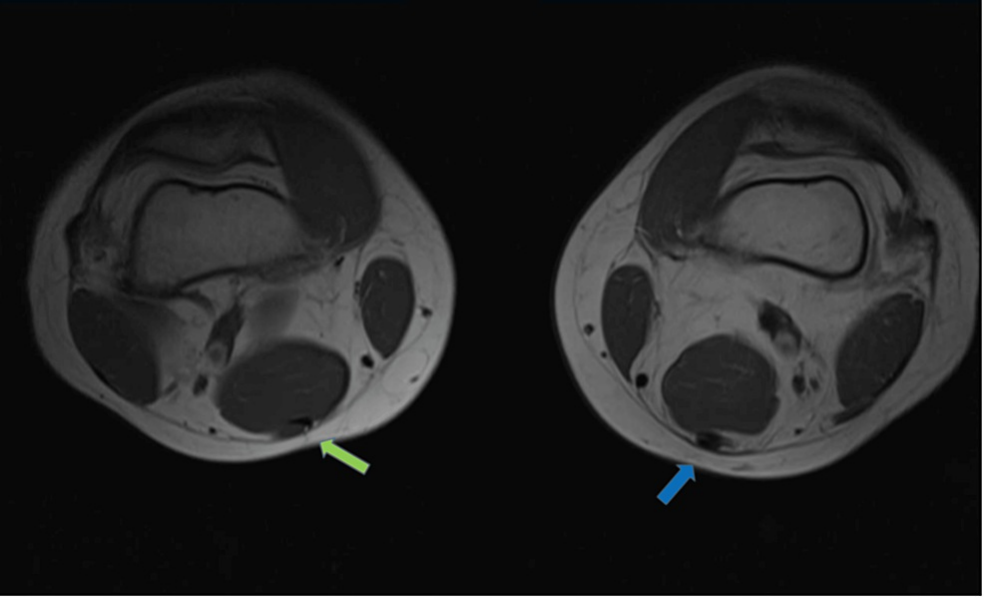
Axial T1 magnetic resonance imaging: Distal third left thigh level. Thickening of the distal third of the left semitendinosus tendon (blue arrow) in comparison to the normal appearance of the right semitendinosus tendon (green arrow).

**Figure 2:**
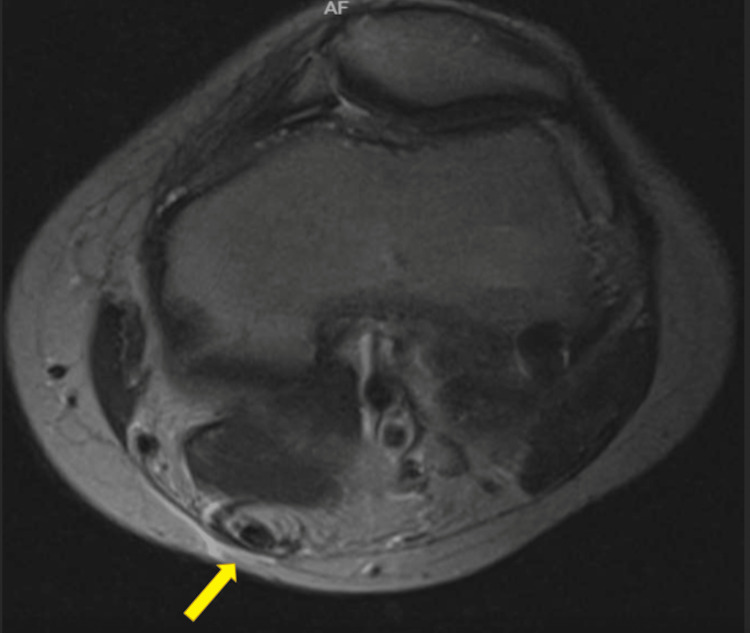
Axial T2 magnetic resonance imaging: Distal third left thigh level. The left semitendinosus tendon (yellow arrow) is slightly thickened and inhomogeneous. A small fluid collection surrounds the tendon.

**Figure 3:**
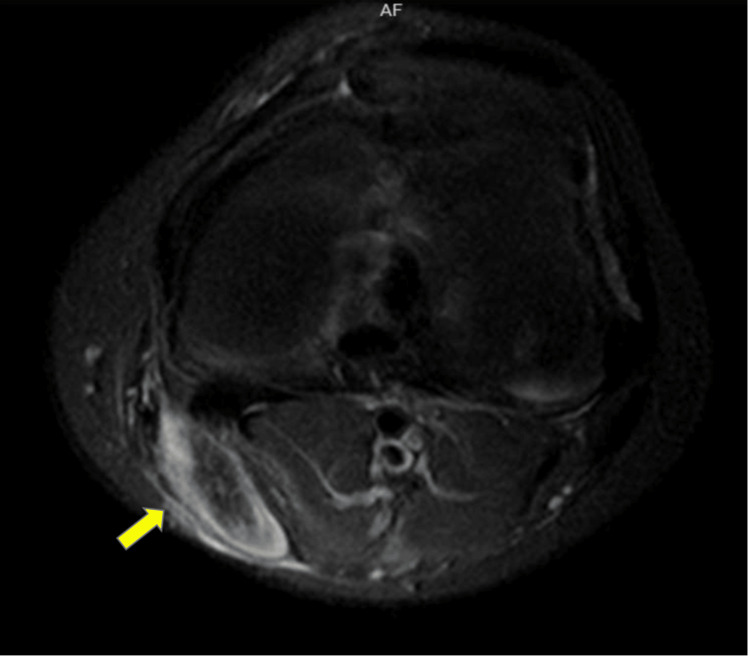
Axial short-T1 inversion recovery magnetic resonance imaging: Left knee level. High signal fluid collection (yellow arrow) surrounding the thickened ruptured semitendinosus tendon, extending into the medial aspect of the upper calf muscle region.

**Figure 4:**
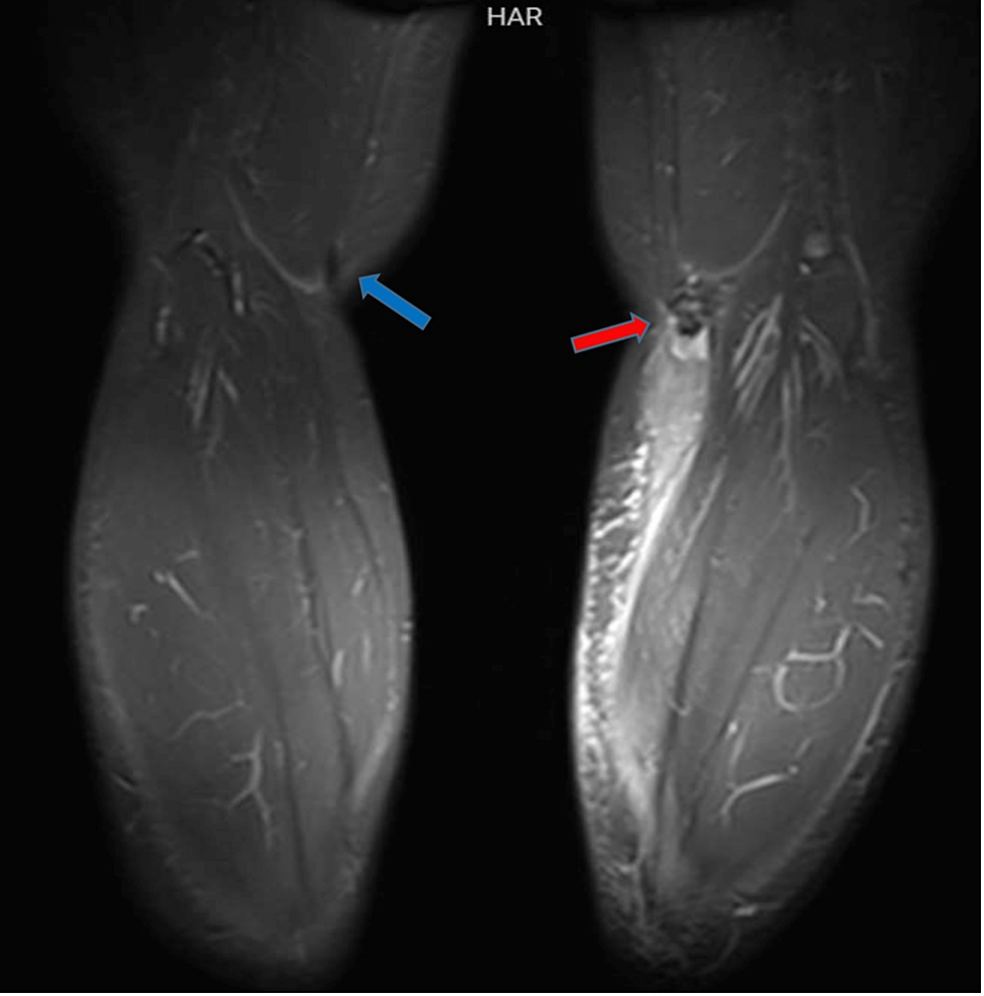
Coronal short-T1 inversion recovery magnetic resonance imaging shows retraction of the ruptured left semitendinosus tendon (red arrow) with high signal fluid collection as well as overlying high signal subcutaneous edematous changes. Note the normal appearance of the right semitendinosus distal tendon (blue arrow).

**Figure 5:**
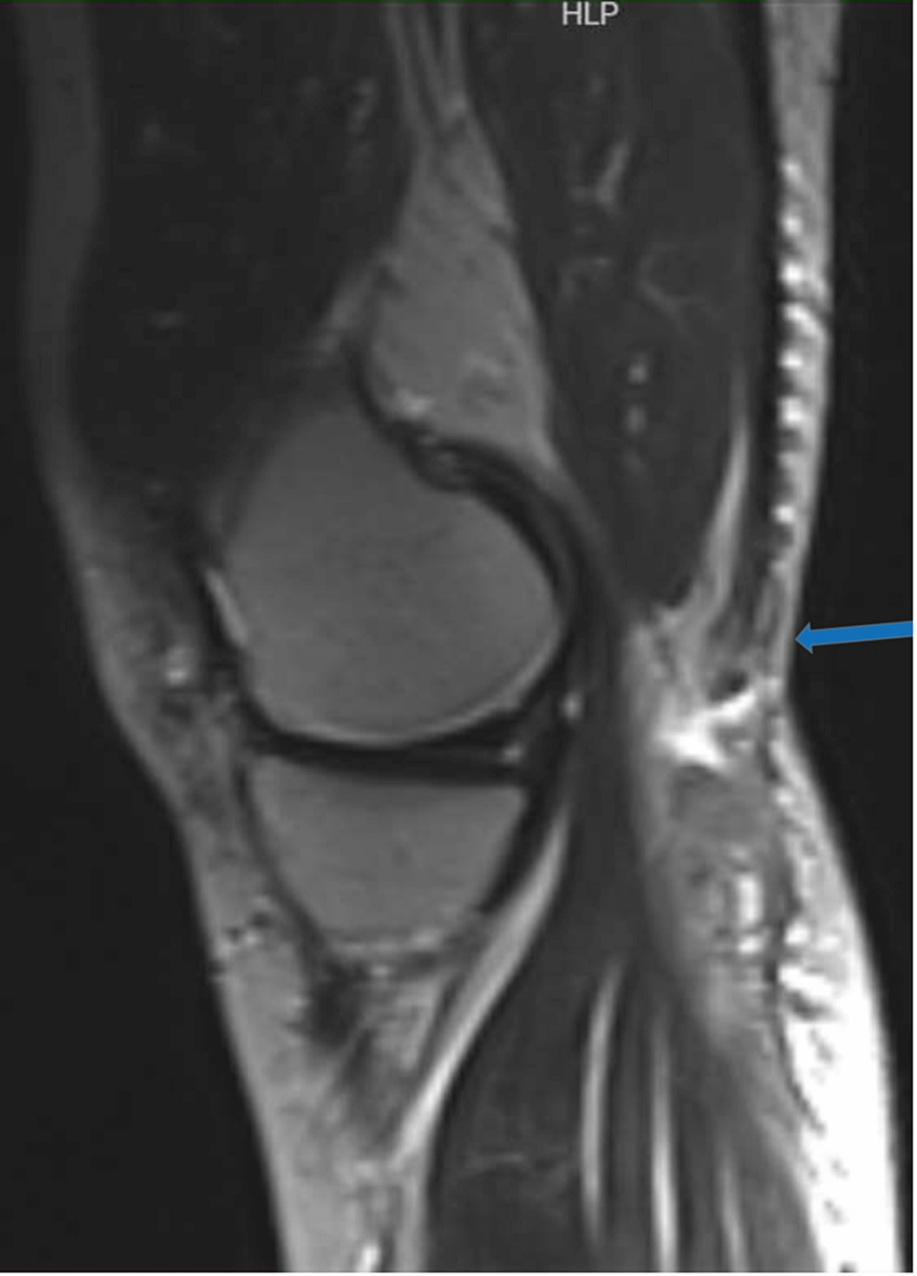
Sagittal T2 magnetic resonance imaging. The left knee joint shows a rupture of the semitendinosus tendon (blue arrow) with distal fluid collection.

A magnetic resonance imaging (MRI) study was conducted 10 days following the trauma to further assess and explore the ST tendon distal rupture. The MRI was performed without contrast, using the following techniques: coronal T1 and T2-weighted images with fat saturation and axial T1 and T2-weighted images. The imaging results demonstrated detachment and retraction of the distal tendon of the ST tendon, with the appearance of inhomogeneous and irregular thickening (Figures 1-5). An extensive fluid collection was also observed circumscribing the tendon and extending along the posteromedial aspect of the medial gastrocnemius muscle, measuring 8.8 × 4.6 × 2.5 cm in its maximum dimension, with evident subcutaneous edema of the surrounding tissue (Figures 4, 5). No abnormalities were found in the muscular compartments or the bones of the calf, and the right distal hamstring tendons appeared normal (Figure 1). The MRI study confirmed the avulsion of the ST tendon and the exclusion of other traumatic injuries, including rupture of the plantaris tendon.

A conservative approach was favored for the treatment of ST tendon avulsion; therefore, the patient was prescribed agents for symptomatic relief. The patient recuperated from his injury with no limitations or restrictions in movement. An overall good prognosis is expected with the avulsion injury to the ST tendon, and full recovery without any subsequential complications is predicted.

## Discussion

The ST muscle is one of the three muscles alongside the biceps femoris and semimembranosus that form the hamstring muscle complex. Arising from the ischial tuberosity, it forms the pes anserinus conjoined tendon that attaches to the anteromedial surface of the proximal tibia [11]. The ST tendon is commonly used as a graft in ACL reconstruction. Pagnani et al. were the first to describe the accessory insertion of the ST separating from the main tendon, which was found in 77% of their cadaveric study subjects [12]. The length and biarticular nature of the ST place it at risk for eccentric load and injury [6,11]. However, distal ST injury remains relatively rare, with a case series of 170 acute hamstring injuries showing only three distal ST avulsions [13].

The most common mechanism of distal ST injury is running or sprinting during competition or training, with the simultaneous contraction of the hamstrings and knee extensors contributing to increased tension across the distal hamstrings [6]. To our knowledge, all cases of distal ST injury occurred during eccentric motions at competitive play or training [11]. However, our paper presents what we believe to be the first case of isolated avulsion of the distal ST tendon due to trauma in a non-athletic environment. We hypothesize that the fall in our patient corresponded to an eccentric overload on the hamstring muscle.

Diagnosis of distal ST tears and avulsions is through a combination of clinical examination with confirmation through imaging. Most patients report acute knee pain with a popping sensation, followed by tenderness at the posteromedial thigh [6]. Further examination tends to show posteromedial knee ecchymosis with weakness in knee flexion [6]. However, imaging plays a crucial role in the confirmation of distal ST injury. MRI has been shown to be more accurate in diagnosing distal hamstring avulsion injuries compared to ultrasound and other imaging techniques [9,13].

MRI has also been found to be more accurate in demonstrating hamstring avulsion injury compared with other imaging modalities, such as ultrasound [1]. The presence of hemorrhage and fluid within the tendon fibrous sheath is an indication of the site of injury, with avulsions appearing as tendon discontinuity with retraction [11,14,15]. MRI provides further utility in differentiating between complete avulsions to partial tears and providing the clinician with the exact location of tendon retraction [11]. It is believed that inadequate MRI techniques contribute to the underreporting of ST injury due to clinical misdiagnosis as distal third muscle belly injury [14]. Cooper et al. proposed a complete visualization of the ST tendon through an MRI of both thigh and knee as a way to circumvent this issue.

No clear consensus has been reached in the literature regarding the optimal management of distal ST injuries, with both conservative and surgical management being used. Conservative management consists of a combination of rest, adequate pain relief, and rehabilitation with range-of-motion and strengthening exercises [6,14].

The ST tendon is commonly harvested in ACL reconstruction, with several studies showing little to no clinically significant loss in long-term knee flexion strength post-ST harvest [10]. Further studies have shown that peak torque values before and after harvest remained unchanged [16], with no significant difference in knee flexion strength compared to patellar tendon harvest [17]. These studies served as the key rationale behind pursuing non-operative management in some studies [10] while also leading Metcalf et al. to conclude that operative repair for ST tears was not critical in achieving optimal outcomes or restoring function [6].

In the surgical management of distal ST avulsion injuries, the choice between surgical repair or tenotomy varies across the literature. As the ST tendon is commonly used as a graft source during ACL reconstruction, tenotomy was chosen over surgical repair in some studies [14,15]. Primary reattachment of the tendon to the tibial bone or reinsertion of the torn tendon with suture repair to the sartorius muscle tendinous portion was also noted as an alternative to tenotomy in some studies [6,11].

In a study by Cooper and Conway comparing the differences between non-operative management with surgical management on 17 athletes with isolated distal avulsion ST injuries, 42% of athletes failed conservative management [14]. The average time of recovery for the conservative management group was 10.4 weeks. Patients who received surgical management immediately within four weeks of injury achieved the fastest recovery time of 6.8 weeks, with the worst recovery time of 29.6 weeks in patients who received surgical management after the failure of conservative management. The authors concluded that early surgery remains an option for patients who prioritize optimizing time to return to sports.

On the other hand, Metcalf et al. showed that athletes treated conservatively had a statistically significant earlier return to sport compared to those who underwent surgical repair, 1.5 ± 0.8 months compared to 3.0 ± 1.3 months with a p-value of 0.002 [6]. However, the nature of surgical management differed between the two studies, with the study by Metcalf et al. opting for reattachment of the distal tendon compared to the tenotomy done in the Cooper and Conway study.

## Conclusions

There is a lack of available evidence to indicate which population subset would benefit from early surgical management as opposed to continuing with conservative management; hence, there is relative uncertainty regarding the optimum management of isolated distal ST avulsion injuries. Therefore, clinical judgment and patient-centered decision-making are essential. To our knowledge, this is the first case of such an injury in a non-athletic patient, which is why we pursued conservative management, as a return to sport was not a critical factor or a priority for this patient.
